# Transparent PC/PMMA Blends Via Reactive Compatibilization in a Twin-Screw Extruder

**DOI:** 10.3390/polym11122070

**Published:** 2019-12-12

**Authors:** Tobias Bubmann, Andreas Seidel, Volker Altstädt

**Affiliations:** 1 Department of Polymer Engineering, University of Bayreuth, Universitätsstraße 30, Bayreuth 95447, Germany; tobias.bubmann@uni-bayreuth.de; 2 Covestro Deutschland AG, Business Unit Polycarbonates, Research & Development, Development Blends, Leverkusen 51365, Germany; andreas.seidel@covestro.com; 3 Bavarian Polymer Institute and Bayreuth Institute of Macromolecular Research; University of Bayreuth, Universitätsstraße 30, Bayreuth 95447, Germany

**Keywords:** transparency, PC, PMMA, blend, transesterification, PC-graft-PMMA copolymer, reactive compounding, continuous reactive extrusion, compatibilization

## Abstract

The effect of different catalysts on reactive compatibilization of 50/50 polycarbonate (PC)/polymethylmethacrylate (PMMA) blends achieved via transesterification that occurs during compounding in a twin-screw extruder was investigated on a phenomenological (optical and mechanical properties), mesoscopic (phase morphology), and molecular level (PC-graft(g)-PMMA-copolymer formation and polymer molecular weight degradation). Formation of PC-(g)-PMMA-copolymer by transesterification resulting in transparent mono-phase PC/PMMA blends with obviously improved compatibility of the two polymer constituents requires use of a suitable catalyst. As a side-effect, PC-(g)-PMMA-copolymer formation by transesterification is always accompanied by a significant simultaneous decomposition of the molecular weight (M_w_) of the PC. For the first time, a colorless, transparent (mono-phase) PC/PMMA 50/50 blend was achieved by a twin-screw extrusion process that can be easily transferred into industrial scale. To achieve this milestone, 0.05 wt% of a weakly acidic phosphonium salt catalyst had to be applied. As a result of the decrease in M_w_ of the PC, the mechanical properties (e.g., tensile strain at break and impact strength) of the obtained blends were significantly deteriorated rather than improved as targeted by the polymer compatibilization; therefore, the produced transparent PC/PMMA blends are considered not yet technically suitable for any industrial applications. Different manufacturing process strategies that do not inherently result in PC degradation as a side effect of PC-graft(g)-PMMA-copolymer formation have to be developed to potentially achieve transparent PC/PMMA blends with a useful balance of properties. Based on the experimental observations of this study, a new mechanism of the transesterification reaction occurring during reactive compounding of PC and PMMA in the presence of the effective catalysts is proposed.

## 1. Introduction

Blending of polymers is a simple, versatile, and economical tool for developing new polymer materials with tailored properties. By combining the benefits of different polymers or even, in favorable cases, leveraging property synergies of the blend partners, polymer blends can fulfill complex requirements of many industrial applications [1,2,3]. For example, the automotive industry plays a key user role in the polymer blend market [4]. Major advantages of tailoring polymer blends, as compared to developing new polymers, are the short time-to-market and typically no investment needed for scale-up, so in essence lower hurdles for successful market introduction of new products.

In particular, polycarbonate (PC)-based blends are of great commercial interest. Beyond the improvement of already industrially established PC blends like PC/acrylonitrile-butadiene-styrene (ABS), PC/styrene-acrylonitrile (SAN), or PC/polybutylene terephthalate (PBT), the development of novel PC blends with so far not yet exploited polymeric blends partners still has enormous scientific, technical, and economic potential. For instance, blending of PC with polymethylmethacrylate (PMMA) is believed to have the potential to allow some technical shortcomings of PC to be overcome, such as scratch sensitivity, chemical resistance, or birefringence, while retaining the appreciated unique benefits of PC, such as good heat and impact resistance. The main challenge of this blend system is the loss of transparency that is typically observed during compounding and is caused by the immiscibility of technical PC and PMMA grades at most composition ratios [5]. A change of the two well-separated glass transition temperatures (*T_g_*) of the individual polymers to a single *T_g_* in the blend as well as a change from an opaque to a transparent blend material has been used as experimental proof of improved miscibility of the blend partners in PC/PMMA blends [6,7].

Different process strategies (e.g., solution casting or melt mixing) have been reported in the literature to obtain transparent or translucent blends. As solution casting [8,9,10,11,12] is not really an industrially relevant process, it will not be further elaborated in the context of the present study. For melt mixing, three fundamentally different strategies have been described in the literature that result in PC/PMMA blends with improved polymer compatibility, namely: a) addition of nanoparticles that accumulate in the polymer interphase and affect interfacial surface tension [13,14], b) use of presynthesized PC-block(b)-PMMA copolymers as an additive for the same purpose [15,16,17], and c) reactive compatibilization by catalytic transesterification of PC and PMMA under in-situ formation of PC-graft(g)-PMMA copolymers [6,7,13,18,19,20,21,22].

Reactive compatibilization by catalytic transesterification is a common strategy in blends manufacturing and has already been described repeatedly for PC blends. For example, it has been applied to PC blends such as PC/PBT [23,24,25] and PC/PET [26,27,28]. For PC/PMMA blends, prior art using this methodology is already existing as well. The finding of a polymer interchange reaction at defined conditions of melt mixing of PC and PMMA in the absence of a catalyst was mentioned for the first time by Rabeony et al. in 1992 [29]. In 1998, Montaudo et al. [18] reported the transesterification of PC/PMMA (70/30) blends via reactive extrusion with residence times from 5 to 60 min in the presence of dibutyltin oxide (SnOBu_2_) as catalyst. Transparency was not achieved by this reactive extrusion process though. Occurrence of a transesterification reaction was not explicitly proven in this study; rather results just indicated a depolymerization of PMMA with a consequent further reaction. Penco et al. [19,20] investigated tetrabutylammonium tetraphenylborate (TBATPB) as transesterification catalyst. Miscible blends, having a single glass transition temperature (*T_g_*), were achieved and evidence of an ester–ester exchange reaction provided via FTIR analysis. However, still no transparent blends could be produced. Singh et al. [6,21] achieved for the first time fully transparent blends of PC and PMMA by using tin(II) chloride dihydrate (SnCl_2_∙2H_2_O) and tin(II) ethyl hexanoate as transesterification catalysts. They observed a single *T_g_* and a high transparency for PC/PMMA blends with different polymer ratios. Furthermore, they postulated the formation of a copolymer based on NMR and FTIR spectroscopy results. Samarium acetyl acetonate was used as a novel catalyst by Bunleechai et al. in 2013 [22]. They proposed a more stable morphology and enhanced mechanical properties but could not support their findings with any analytical characterization of the produced PC/PMMA materials. Recently, two patent applications [30,31] were also published, claiming zinc acetate and dibutyl tin dilaurate as suitable transesterification catalysts for reactive compatibilization of PC and PMMA.

The literature from Singh et al. as well as the two patent filings suggest that good transparency can only be achieved with a quite high (and thus detrimental) amount of catalyst. However, both studies could not demonstrate that a miscible and transparent PC/PMMA blend is achievable at a ratio of 50/50 (wt%/wt%). Furthermore, none of the previous work reported material properties beyond transparency (e.g., mechanical performance), which are of utmost importance for industrial application of any such material. Rather, they all focused on microscale melt mixing in discontinuous compounding aggregates using large residence times. Transparent PC/PMMA blends produced in a continuous extrusion process with much shorter residence times, typically in the range of 30–60 s, had not been reported up to now although such processes are economically preferred and can be more easily scaled-up.

In the present study, we thus targeted: (1) the identification of an optimized, more efficient transesterification catalyst, (2) the transfer of the so far academic microscale discontinuous melt mixing approach to an industrially more relevant, easily to scale-up continuous twin-screw extrusion process and, in addition, (3) a better mechanistic understanding of the chemistry of the PC/PMMA transesterification that occurs during reactive compounding of the two polymers in the presence of effective catalysts. As the PC/PMMA 50/50 composition had not yet been reported in previous work to result in transparent material and thus seems most challenging from the scientific point of view, we decided to use this particular PC/PMMA ratio for our case study. Moreover, we aimed assessment of a meaningful properties profile that allows more reliable conclusions than the previous scientific work on the potential relevance of the obtained polymer blends for targeted industrial applications, e.g., in the automotive industry.

## 2. Materials and Methods

### 2.1. Materials

PC (Makrolon^®^ 2408) was obtained from Covestro Deutschland AG (Leverkusen, Germany), and PMMA (Plexiglas^®^ 8H) was purchased from Evonik Industries AG (Essen, Germany). The molecular weights of these raw materials measured by gel permeation chromatography (GPC), both with polystyrene standard, were M_w_ = 46,000 g/mol and M_n_ = 27,000 g/mol for the PC and M_w_ = 130,000 g/mol and M_n_ = 74,500 g/mol for the PMMA. The PC and PMMA grades were chosen based on a prescreening of commercially available grades of these polymer classes, such that the blend partners under reactive compounding conditions (i.e., 260 °C and shear rates of about 100 s^−1^) exhibited similar melt viscosities. This was done with the rationale that a viscosity ratio PC/PMMA of about 1 will result in a most efficient melt mixing. Both PC and PMMA raw materials were predried in vacuum at 60 °C before compounding. The PC used for the blending experiments (Makrolon^®^ 2408) exhibited a negligible phenolic hydroxyl (pOH) group content, determined by ^1^H-NMR spectroscopy, of <100 ppm. The applied catalysts are listed in Table 1. Based on the literature and patent survey, tin(II) chloride dihydrate (SnCl_2_∙2H_2_O) [6] and zinc acetate [30,31] were selected as state-of-the-art benchmarks. Additionally, a weakly acidic phosphonium salt was investigated as a possible transesterification catalyst (1 g of this catalyst dispersed under steady stirring in 150 mL deionized water of pH = 5.8 yielded an aqueous dispersion with a pH of 4.8).

### 2.2. Experimental

#### 2.2.1. Compounding

PC/PMMA 50/50 blends (50 wt% PC + 50 wt% PMMA) were prepared by melt extrusion using different size twin-screw extruders. A discontinuously running micro compounder (MC)15 from Xplore (Sittard, Netherlands) was used with residence times in the range of 3 up to 30 min. Compounding conditions were set at a melt temperature of 260 °C and a rotation speed of 100 rpm. For the continuous reactive extrusion, a Process 11 parallel twin-screw extruder from Thermofisher Scientific (Waltham, MA, USA) and a ZSK26 MC18 corotating twin-screw extruder from Coperion (Stuttgart, Germany) were used at melt temperatures of 260 °C and residence times of about 90 s and 30 s, respectively. The blend components were grounded with a Retsch ZM 100 Ultra Mill (Haan, Germany), and the resulting polymer powders were homogeneously mixed with the catalysts prior to melt mixing. Catalyst contents were chosen in the range of 0.05 to 0.3 wt%.

#### 2.2.2. Thermal Analysis

Differential scanning calorimetry (DSC) from Mettler Toledo (Columbus, OH, USA) was carried out to investigate the change in glass transition temperatures (*T_g_*) of the different PC/PMMA blends. The measurements were done under nitrogen atmosphere at a heating/cooling rate of 10 K/min. The samples were heated from 0 to 200 °C, held at 200 °C for 3 min, and then cooled down to 0 °C. This depicted the first run. For the determination of the *T_g_* the 2^nd^ heating curves were used, which consist of the same temperature program as the 1^st^ run.

#### 2.2.3. Fourier Transform Infrared Spectroscopy (FTIR) Analysis

FTIR was done to proof and semiquantitatively estimate extent of formation of PC-g-PMMA copolymer as a result of transesterification upon compounding of the PC/PMMA blend compositions. For this purpose, 5 g of the compounded PC/PMMA blends were extracted in 100 mL of acetone under stirring for 24 h at room temperature, followed by filtration with a Büchner funnel to separate the acetone insoluble part of the blend (containing mostly PC) from the acetone soluble part (containing mostly PMMA). Afterwards, the materials were dried to remove the residual acetone. The obtained fractions of the products were analyzed separately by FTIR with an FTIR spectrometer Nexus 470 from Nicolet (Thermofisher Scientific) (Waltham, MA, USA) in attenuated total reflection (ATR) mode. Spectra were recorded in the range of 400–4000 cm^−1^ with a resolution of 1 cm^−1^. As a methodology validation, a physical mixture of PC/PMMA 50/50 produced by melt compounding in absence of any catalyst was investigated by this procedure and a complete separation of the two blend components demonstrated. I.e., the acetone soluble part only consisted of pure PMMA, and the acetone insoluble part only consisted of pure PC.

#### 2.2.4. Nuclear Magnetic Resonance Spectroscopy (NMR)

^1^H-NMR was used to quantify the content of PC-g-PMMA formation during reactive extrusion of PC and PMMA in presence of 0.05 wt% of the phosphonium salt catalyst. For this purpose, ^1^H-NMR spectra of the neat PC and PMMA raw materials as well as the acetone insoluble part of the PC/PMMA 50/50 blend were recorded. In each case, 30 mg of the investigated material was completely dissolved in about 0.8 mL deuterated chloroform. The NMR spectra were recorded with a Bruker Avance (Billerica, MA, USA) with 300 MHz at room temperature.

#### 2.2.5. Gel permeation chromatography (GPC)

For molecular weight distribution determination gel permeation chromatography (GPC) measurements of the neat PC and PMMA raw materials as well as of the acetone soluble and insoluble parts of the compounded blends were performed on an instrument having four PSS-SDV gel columns (particle size = 5 µm) with a porosity range from 102 to 105 Å (PSS, Mainz, Germany) using a nonselective refractive index detector (Shodex; Techlab, Japan). Tetrahydrofuran (THF) was used as the solvent for the respective polymer fractions and as the eluting solvent. The sample was filtered through a 0.22 µm PTFE filter after solving in THF and before GPC analysis. Eluent flow rate was set at 1.0 mL/min. The calibration was done with narrowly distributed polystyrene (PS) homo-polymer.

#### 2.2.6. Optical Properties

Transparency and color were qualitatively assessed by visual inspection of 1 mm thick hot-melt pressed specimens. Furthermore, haze and yellowness indexes were determined according to DIN6167 (1980 version) “Description of Yellowing of Nearly White or Nearly Colorless Materials” on test specimens of dimension 60 mm × 40 mm × 4 mm that had been injection molded at a melt temperature of 260 °C.

#### 2.2.7. Mechanical and Rheological Properties

Tensile testing was performed at room temperature in accordance to ISO 527 (1996 version). Injection molded dog-bone specimens (170 mm × 10 mm × 4.0 mm) were tested at a rate of 50 mm/min and at 1 mm/min for Young’s modulus measurement. Impact testing was performed at room temperature according to ISO 180/1U (2013 version) using injection molded specimens of dimension 80 mm × 10 mm × 4 mm. Heat resistance is reported as Vicat B/120 values, which were determined on injection molded test specimens of dimension 80 mm × 10 mm × 4 mm according to ISO 306 (2014 version). Melt viscosity was determined in accordance to ISO 11443 (2014 version) at a melt temperature of 260 °C. Melt viscosities have been recorded at various shear rates in the range of 50 to 5000 s^−1^. Test specimens were injection molded at a melt temperature of 260 °C.

#### 2.2.8. Transmission electron microscopy (TEM)

The morphological characterization was performed via bright field transmission electron microscopy (TEM) using a Zeiss EM922 OMEGA (Oberkochen, Germany) at an acceleration voltage of 200 kV. Ultrathin sections (~50 nm) were prepared from the compound pellets using an ultramicrotome Leica EM UC7 (Wetzlar, Germany). The ultrathin sections were stained with ruthenium tetroxide (RuO_4_) for 15 min in order to enhance the contrast of the constituent polymers.

## 3. Results and Discussion

### 3.1. Compounding in a Laboratory-Scale Discontinuous Extrusion Process

In the first experiments, we reproduced, for comparison reasons, the compounding process as reported by Singh et al. [6] using the discontinuous micro compounder (sample size: 15 g) and SnCl_2_∙2H_2_O as the reference catalyst. In contrast to the work by Singh, the axial force of the extruder housing was continuously monitored to investigate viscosity changes of the melt composition [32] that can be indicative of any reactions (e.g., polymer decomposition and transesterification) occurring during compounding.

**Figure 1:**
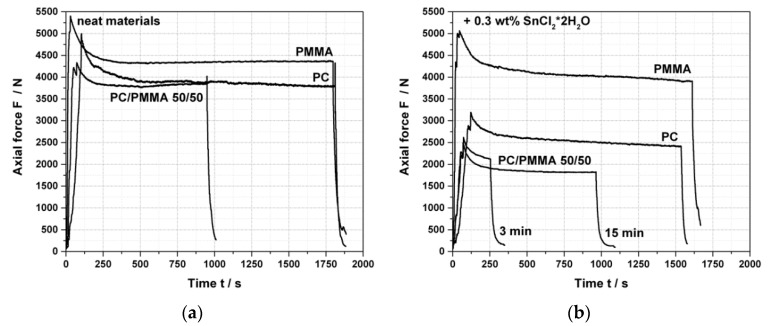
Force vs. time curves of (**a**) neat polycarbonate (PC), neat polymethylmethacrylate (PMMA), and PC/PMMA 50/50 in the absence of any catalyst and (**b**) of the same materials in presence of 0.3 wt% SnCl_2_∙2H_2_O catalyst.

The neat polymer components as well as the PC/PMMA 50/50 blend were melt mixed in absence of catalyst (Figure 1a) and in presence of 0.3 wt% of SnCl_2_∙2H_2_O (Figure 1b). The according axial force vs. time curves are shown in Figure 1. The PC/PMMA 50/50 blend produced in absence of a catalyst was opaque, while the according blend produced in presence of the catalyst was transparent but exhibited a strongly brownish color. This is in contrast to the results by Singh et al. [6], who reported that blends of this 50/50 composition were opaque.

Generally, axial force changes during the first up to 500 s (initial increase and temporary decrease afterwards) are related to the filling of the extruder and melting of the polymer components (Figure 1). In Figure 1b, in addition they can be potentially related to any instantaneous melt reaction and any potential softening effect due to the used catalyst. Any further changes of the axial force occurring after the first 500 s have to be related to slower reactions (e.g., polymer degradation and/or transesterification). In case of no such reaction, a steady state (constant axial force) is expected.

The axial forces observed for the neat polymer components and the PC/PMMA 50/50 blend in absence of a catalyst (Figure 1a) are pretty constant at > 500 s and at a similar level within repeatability of this measurement for PC, PMMA, and the PC/PMMA 50/50 blend. This is expected based on the similar melt viscosities of the chosen polymers.

While the axial force observed for the pure PMMA is hardly affected by the catalyst, the addition of 0.3 wt% of SnCl_2_∙2H_2_O leads to an instantaneous decrease in the axial force of the pure PC and an even more pronounced decrease in case of the PC/PMMA blend (compare Figure 1a,b). The latter finding turns out reproducible as the two experiments with different residence times are showing. Interestingly, as a consequence, the axial force observed for the PC/PMMA blend produced with the catalyst is even lower than the axial forces of both neat blend partners when compounded with the catalyst (Figure 1b). Our interpretation is that the catalyst likely degrades the PC molecular weight (M_w_), and this polymer degradation is even more pronounced if PMMA is present. This can be considered as a first potential experimental indication of a transesterification reaction occurring between PC and PMMA in the presence of SnCl_2_∙2H_2_O as catalyst, as the transesterification according to the mechanism proposed by Singh et al. [6] will always result in a chain scissoring of the PC.

However, based on this single experiment alone, the interpretation is still speculative. An alternative possible explanation of this experimental observation (which, in our view of the full picture of our further experimental findings to be presented in the following, is much less likely) is that the SnCl_2_∙2H_2_O is unequally distributed in the PC/PMMA blend and accumulates in the PC phase. This could explain both the observed inactivity of the catalyst in terms of PMMA degradation and also the stronger reduction of axial force in the PC/PMMA 50/50 blend compared to both the pure PC and pure PMMA. The rationale is: if the catalyst was indeed accumulated in the PC phase, the effective concentration in the PC would be higher (actually, in a PC/PMMA 50/50 composition, double the amount), and thus PC degradation induced by that catalyst is expected to be more pronounced than if the same amount of catalyst was added to pure PC.

### 3.2. Compounding in a Laboratory-Scale Continuous Extrusion Process

To the best of our knowledge, the reactive extrusion of PC/PMMA blends in presence of a transesterification catalyst via a continuous extrusion process up to now has not yet been reported in the scientific or patent literature. The differences of such continuous extrusion processes compared to the discontinuous experiments reported previously are lower residence times (30–90 s compared to 5–60 min) on the one hand and higher shear forces (superior mixing) during compounding on the other. In the initial continuous extrusion experiments that targeted fundamental investigation of the effects of the different catalysts on optical properties (Chapter 3.2.1), phase morphology (Chapter 3.2.2), as well as transesterification and polymer degradation (Chapter 3.3.3), the small-scale twin-screw extruder (Process 11) was used with a residence time of 90 s.

#### 3.2.1. Influence of Catalysts on Optical Properties (Visually Phenomenological Effects)

**Figure 2:**
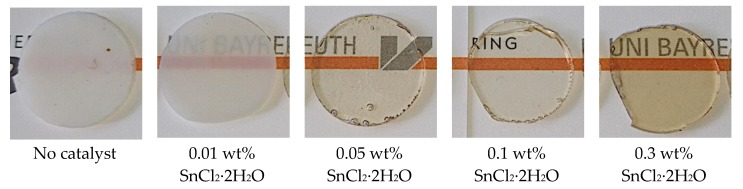
Influence of SnCl_2_∙2H_2_O catalyst content on the transparency and color of the PC/PMMA 50/50 blend produced in a continuous extrusion process (Process 11).

First, we investigated the effect of the catalyst content on the achieved transparency of the polymer blends, in order to define an optimum concentration of the catalyst to be used in the further catalyst screening. For this purpose, we tested the benchmark catalyst SnCl_2_∙2H_2_O [6] at concentrations at 0.01, 0.05, 0.1, and 0.3 wt%. Figure 2 shows 1 mm thick hot-melt pressed specimens of the produced PC/PMMA 50/50 blends.

The PC/PMMA 50/50 blend produced in absence of any catalyst turns out opaque. Already the addition of only 0.01 wt% SnCl_2_∙2H_2_O leads to a slightly translucent blend, which becomes transparent at an increased catalyst concentration of 0.05 wt% and above. While transparency is not further improved at catalyst contents above 0.05 wt%, the color of the blends turns increasingly brownish. The steady-state torque of the extruder in the compounding process decreases with catalyst addition. This is consistent with the results of the axial force changes previously observed in the discontinuous microcompounder experiments upon catalyst addition.

**Figure 3:**
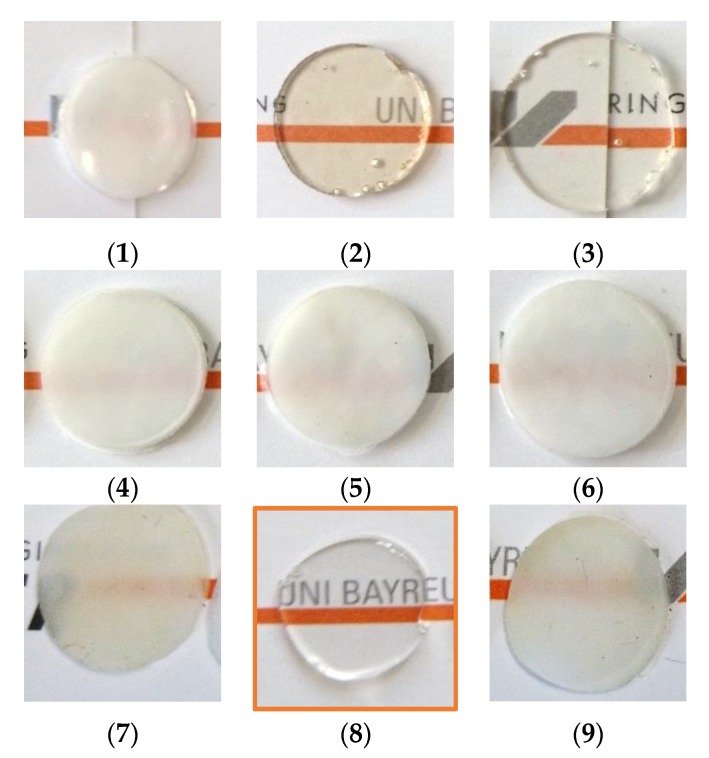
PC/PMMA 50/50 blends (**1**) without any and with 0.05 wt% of catalyst (**2**) SnCl_2_∙2H_2_O, (**3**) anhydrous SnCl_2_, (**4**) tin(II) acetate, (**5**) tin(II) ethyl hexanoate, (**6**) tin(II) acetylacetonate, (**7**) zinc acetate, (**8**) phosphonium salt, and (**9**) zinc acetylacetonate produced by a continuous extrusion process (Process 11).

As the increase in color at higher catalyst contents is undesired for industrial applications of the blends, we decided to use a catalyst content of 0.05 wt%. At this content, the benchmark catalyst provided the optimum balance of high transparency and lowest possible color for the further catalyst screening study. In this screening study, we used SnCl_2_∙2H_2_O and zinc acetate as reference catalysts already reported in the scientific and patent literature, respectively, for transesterification of PC and PMMA. Starting from these two references and considering other knowledge about substances that are in principal useful transesterification catalysts for different purposes, we systematically varied cation, anion, and hydration water content in the choice of the investigated catalysts in order to allow assessment of the effects of those parameters (Table 1). In addition, a weakly acidic phosphonium salt was added to the catalyst screening. Figure 3 shows 1 mm thick hot-melt pressed specimens of the PC/PMMA 50/50 blends produced with the different catalysts.

Of the investigated catalysts only the SnCl_2_∙2H_2_O reference catalyst (Figure 3(2)), the anhydrous SnCl_2_ (Figure 3(3)), and the phosphonium salt (Figure 3(8)) resulted in transparent blends. However, while the blends produced with both tin chloride catalysts show brownish color, only the phosphonium salt resulted in a completely transparent and, at the same time, colorless blend. Torque of the extruder was reduced for all the compositions resulting in transparent blends compared to the compositions produced with catalysts resulting in opaque blends.

The second catalyst, namely tin(II) ethyl hexanoate, reported by Singh et al. [21] to result in transparent PC/PMMA 80/20 blends when used at a content of 0.5 wt% in the discontinuous extrusion process that he investigated, did not result in a transparent PC/PMMA 50/50 blend at the lower catalyst content of 0.05 wt% in the continuous extrusion process used here (Figure 3(5)). Additionally, zinc acetate disclosed as catalyst for transesterification of PC and PMMA in the patent literature [30,31] did not result in transparent blends under our experimental conditions (Figure 3(7)). However, this is in line with the fact that the claims of that patent did actually exclude the PC/PMMA ratio of 50/50 that is used in our current study.

#### 3.2.2. Influence of the Catalysts on Phase Morphology and Polymer Miscibility (Mesoscopic Level Effects)

**Figure 4:**
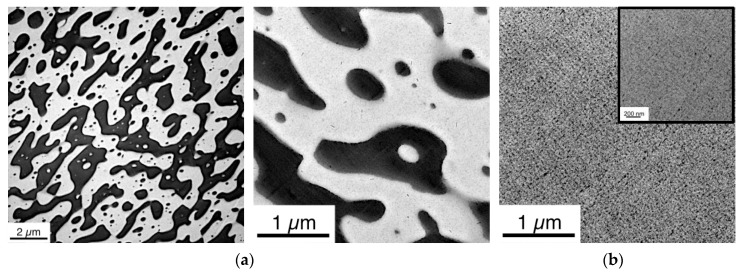
Transmission electron microscopy (TEM) images of PC/PMMA 50/50 blends produced (**a**) in absence of a catalyst (opaque) at two different magnifications and (**b**) in presence of 0.05 wt% of phosphonium salt (transparent).

The transparency can be considered as an indicator for a fully miscible PC/PMMA blend. To proof this interpretation, TEM and DSC investigations were performed. Figure 4 shows a comparison of the TEM images of the PC/PMMA 50/50 blends produced in absence of a catalyst (Figure 4a, opaque) and in presence of 0.05 wt% of the phosphonium salt (Figure 4b, transparent).

While the opaque blend produced in absence of any catalyst is showing phase separation of PC and PMMA resulting in a cocontinuous morphology that is actually expected at the 50/50 ratio of immiscible blend partners (phase domain sizes in the range of several microns), the transparent blend produced with the phosphonium salt clearly shows only a single phase. Even at increased magnification, no phase separation can be detected. We consider this finding as very strong indication that complete miscibility of the two polymers has been achieved in this blend as a consequence of use of this specific catalyst at the chosen concentration. If there was any potential remaining phase separation that cannot be detected at the maximum magnification used in our TEM investigations, the resulting phase domain sizes would have been at least dramatically decreased versus the blend produced in absence of any catalyst (i.e., down to sizes <10 nm, which is below the half of the wavelength of visible light and thus explains the observed transparency of the according blend).

**Figure 5:**
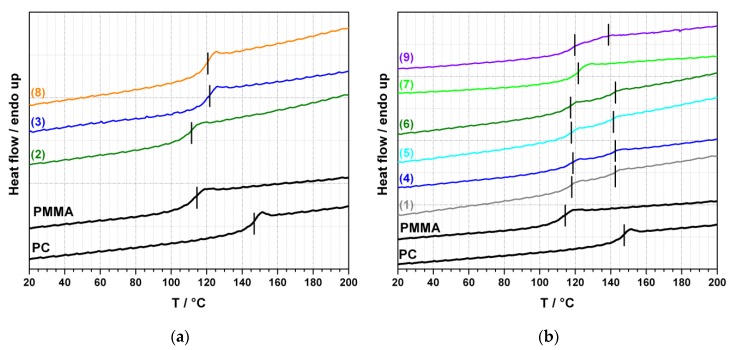
(**a**) Differential scanning calorimetry (DSC) thermograms (2^nd^ heating) of pure PC, pure PMMA, and of the transparent PC/PMMA 50/50 blends produced in presence of 0.05 wt% of catalysts SnCl_2_∙2H_2_O (2), anhydrous SnCl_2_ (3), and phosphonium salt (8); (**b**) DSC thermograms of pure PC, pure PMMA, and of the opaque PC/PMMA 50/50 blends produced in absence of any catalyst (1) and in presence of 0.05 wt% of catalysts tin(II) acetate (4), tin(II) ethyl hexanoate (5), tin(II) acetylacetonate (6), zinc acetate (7), and zinc acetylacetonate (9).

The results of DSC of the transparent (Figure 5a) and opaque blends (Figure 5b) are in line with this interpretation of the TEM images. The DSC curves of all transparent blends show only a single *T_g_* as is expected in case of a completely miscible polymer blend. In contrast, the DSC curves of the opaque blends, with the exception of the blend produced with zinc acetate, show two well-separated glass transitions in line with the presence of a two-phase blend system. The values of the single *T_g_*’s of the transparent PC/PMMA blends are; however, shifted towards lower temperatures compared to the values expected at the 50/50 ratio of blend partners from Fox equation [33]. This can be explained by degradation of the PC M_w_ (see GPC measurement below) during compounding in the presence of those catalysts resulting in transparent blends, which results in a significant reduction of the glass transition temperature of the PC component. Such PC degradation is indicated by lower axial force (in the micro compounder experiments) and lower steady-state torque (in the continuous extrusion experiments) observed during compounding of the transparent vs. the opaque PC/PMMA blends of the same 50/50 ratio.

From the opacity of the blend produced with zinc acetate, it obviously has to be concluded that this material exhibits a two-phase morphology. The single glass transition observed in the DSC in this case is thus not a consequence of complete polymer miscibility but can be considered rather to be related to a reduction of the *T_g_* of the PC caused by PC degradation to a value that cannot anymore be separated in the DSC from the *T_g_* of the PMMA. An alternative interpretation is that there is still a small percentage of immiscible blend partners in the material, which is not detectable by DSC (single *T_g_*) but still is detectable by light scattering (opacity).

#### 3.2.3. Influence of the Catalysts on Transesterification and Polymer Degradation (Molecular Level Effects)

Complete polymer miscibility that results in transparent PC/PMMA blends when selected catalysts are used in the compounding process can be the consequence of PC-g-PMMA copolymer formation by transesterification. Such copolymer acting as compatibilizer was proposed by Singh et al. [6]. Alternatively, however, polymer miscibility can also be the consequence of degradation of one or both polymer blend partners during compounding using such selected catalysts or a consequence of a combination of both polymer degradation and PC-g-PMMA copolymer formation. That is because blends of semimiscible polymers A and B are known to transit from two-phase to one-phase morphology if 1/N_A_ + 1/N_B_ exceeds a threshold value [34]. This threshold is a constant for a given polymer pair and temperature. N_A_ and N_B_ are the weight average numbers of monomer units in polymers A and B, respectively. In other words, two semimiscible polymers A and B become fully miscible if the molecular weights of polymers A and/or B are small enough. The disadvantage of miscible blends resulting from reduced molecular weight of either blend partner is that the low M_w_ of the polymer(s) will typically result in negative impacts on the mechanical material performance of the blend. As the next step, we thus investigated both PC-g-PMMA copolymer formation and polymer degradation occurring during continuous compounding of PC/PMMA 50/50 blends in the presence of the various catalysts. The target was to identify the actual root cause of the polymer miscibility in the transparent blends obtained with selected catalysts.

**Figure 6:**
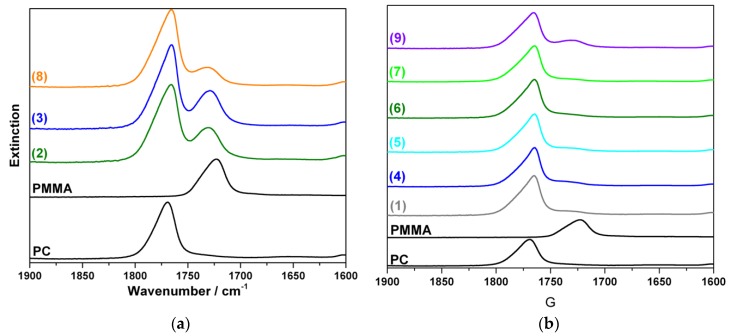
(**a**) FTIR spectra of pure PC, pure PMMA, and the acetone insoluble portions of the transparent PC/PMMA 50/50 blends produced in presence of 0.05 wt% of catalysts SnCl_2_∙2H_2_O (2), anhydrous SnCl_2_ (3), and phosphonium salt (8); (**b**) FTIR spectra of pure PC, pure PMMA, and the acetone insoluble portions of the opaque PC/PMMA 50/50 blends produced in absence of any catalyst (1) and in presence of 0.05 wt% of catalysts tin(II) acetate (4), tin(II) ethyl hexanoate (5), tin(II) acetylacetonate (6), zinc acetate (7), and zinc acetylacetonate (9).

To prove formation of and quantify any PC-g-PMMA copolymer resulting from transesterification reaction during PC/PMMA compounding, the acetone insoluble portions of the produced blends were investigated by FTIR and ^1^H-NMR measurements. The collected FTIR spectra are shown in Figure 6.

In the FTIR spectra of the acetone insoluble portions of all transparent blends, two clearly distinct carbonyl stretching vibration bends at 1720 cm^−1^ (assigned to PMMA) and at 1770 cm^−1^ (assigned to PC) are observed. A blend fraction that is not soluble in acetone, but nevertheless contains a significant amount of PMMA is strong indication for formation of a PC-g-PMMA copolymer by transesterification in these materials. PMMA that is not chemically bonded to PC had been proven to be completely soluble in acetone under the applied conditions (see experimental part above). On the other hand, the FTIR spectra of the acetone insoluble fractions of the opaque blends (Figure 6b) show essentially only the bend at 1770 cm^−1^, i.e., this fraction of the opaque blends contains essentially only polycarbonate. Our conclusion is that the opaque PC/PMMA blends actually do not contain any significant amounts of PC-g-PMMA copolymer, i.e., practically no transesterification occurred during their compounding. As a proof of this interpretation, FTIR investigations have also been performed on the acetone soluble fractions of the opaque PC/PMMA blends. All spectra exhibited only a single carbonyl stretching vibration bend at 1720 cm^−1^ related to PMMA, so did not provide any indication for presence of PC-g-PMMA copolymer with potentially lower block length of the PC that could be imagined to be soluble in acetone. The opaque material produced with zinc acetylacetonate as catalyst (Figure 6b(9)) likely contains a minor fraction of PC-g-PMMA copolymer as indicated by the small shoulder at 1720 cm^−1^ in the respective FTIR spectrum of its acetone insoluble fraction. Obviously, the amount of the PC-g-PMMA copolymer generated via transesterification in the presence of zinc acetylacetonate is; however, insufficient to result in a transparent blend—at least at the molecular weights of the PC and PMMA resulting in this particular blend upon compounding.

**Figure 7:**
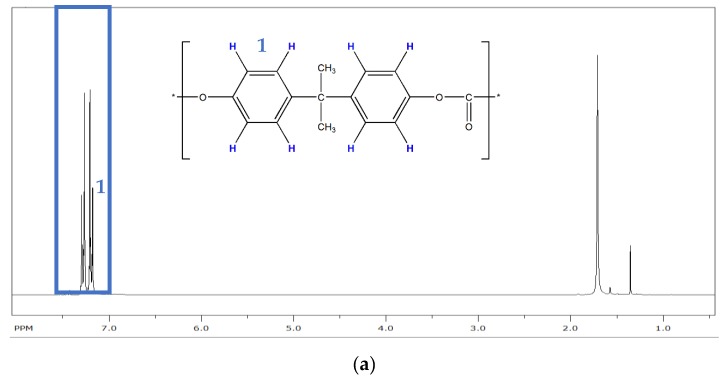

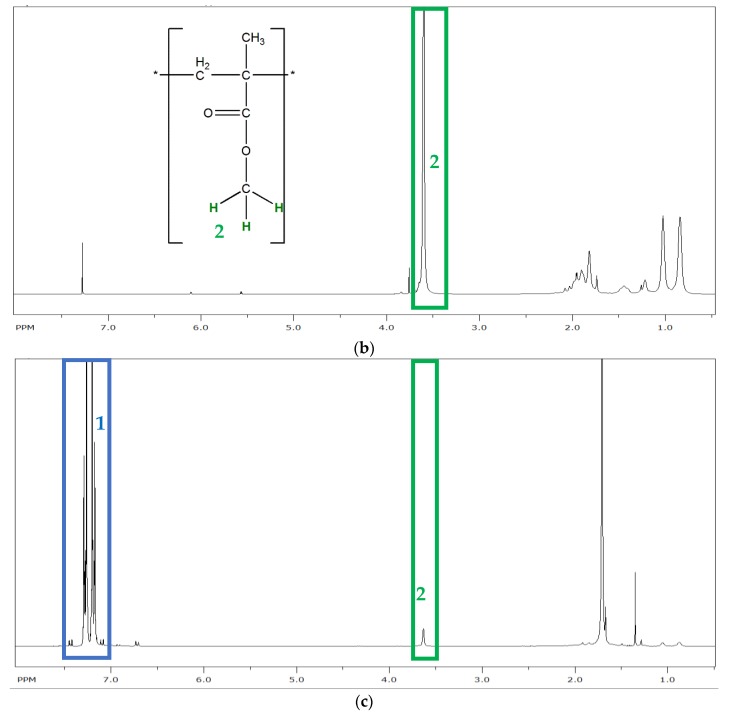
^1^H-NMR spectra of the pure PC (**a**) and PMMA (**b**) raw materials as comparison and of the acetone insoluble part of the transparent PC/PMMA 50/50 blend produced in presence of 0.05 wt% of phosphonium salt (8) (**c**).

FTIR is easy and comfortable to provide semiquantitative proof of PC-g-PMMA copolymer formation because it can be applied on a solid, i.e., solving of the sample prior to investigation is not required. However, FTIR is not the best method for quantification of the extent of the PC-g-PMMA copolymer formation since it requires a calibration and is not very precise due to overlapping IR bends related to PC and PMMA (see Figure 6). We thus decided to do, in addition, quantitative ^1^H-NMR spectroscopy on the acetone insoluble part of the PC/PMMA 50/50 blend produced in the presence of 0.05 wt% of the phosphonium salt catalyst. This sample was chosen as an example because it had displayed the best performance regarding transparency and color (Figure 3). Figure 7 shows the recorded NMR spectrum of this sample as compared to the NMR reference spectra of the neat PC and PMMA feedstock polymers. For the quantitative analysis, the NMR signals at 7.1–7.3 ppm attributed to the eight aromatic protons of the bisphenol-A units in the PC and at 3.6 ppm attributed to the three methyl ester protons of the PMMA were integrated. A content of 10 mol% of PMMA repetition units, corresponding to 4 wt% of PMMA as part of a PC-g-PMMA copolymer was calculated to be present in the acetone insoluble part of the investigated PC/PMMA 50/50 blend. No significant bend at 1770 cm^−1^ attributed to PC had been observed in the acetone soluble part of this blend, so we conclude that no PC-g-PMMA copolymer was extracted by the acetone. We thus estimate that the total content of PC-g-PMMA copolymer that has formed upon reactive compounding in the original PC/PMMA 50/50 blend via transesterification with the phosphonium salt catalyst is in the order of about 2 wt%.

Although the results of the FTIR and ^1^H-NMR investigations confirm presence of PC-g-PMMA copolymer in the transparent PC/PMMA blends, this does not necessarily mean that this copolymer is (exclusively) responsible for the observed transparency. The mechanism of transesterification as proposed by Singh et al. [6] results in scissoring of one PC chain per formed PC-g-PMMA copolymer molecule. Although Singh did not explicitly report according experimental results, such transesterification, hence, must result in a significant reduction of PC M_w_ as a side effect, which actually might be the real root cause, or at least a secondary prerequisite beyond the PC-g-PMMA copolymer formation, for obtaining transparent PC/PMMA blends.

**Figure 8:**
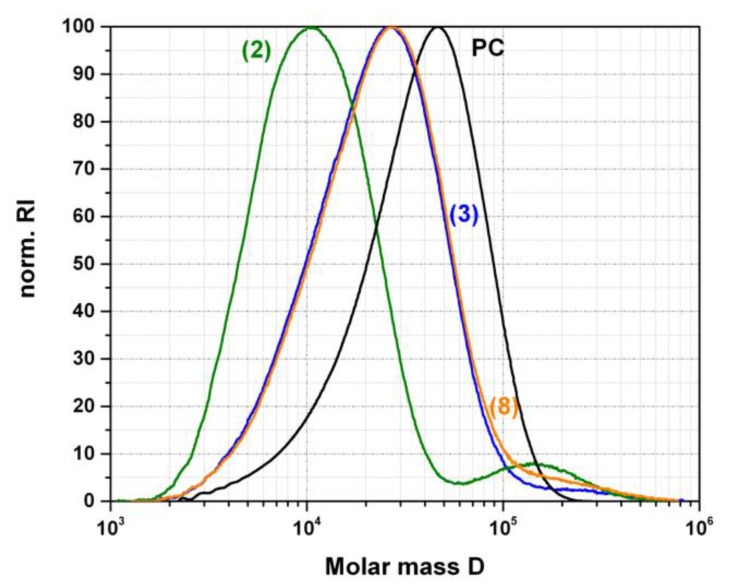
Molecular weight distribution curves measured by GPC of the pure PC raw material as comparison and of the acetone insoluble parts of the transparent PC/PMMA 50/50 blends produced in presence of 0.05 wt% of catalysts SnCl_2_∙2H_2_O (2), anhydrous SnCl_2_ (3), and phosphonium salt (8).

In order to allow judgement about the real root cause of the transparency, we thus performed GPC measurements. Molecular weight distributions determined on the acetone insoluble parts of the transparent PC/PMMA blends are shown in Figure 8. Furthermore, the molecular weights at the peak (maximum) position of the molecular weight distribution, M_peak_ determined for the acetone insoluble parts of both the transparent and opaque PC/PMMA 50/50 compounds are summarized in Table 2. Molecular weight distributions determined on the acetone soluble parts of the transparent blends were essentially identical with the molecular weight distribution of the pure PMMA raw material. This acetone soluble part of a PC/PMMA blend, based on our previous investigation on a physical PC/PMMA mixture compounded in the absence of any catalyst (see experimental part), can be assigned to the unreacted PMMA portions within the reactively compounded products. It thus can be concluded that no degradation of the PMMA polymer has occurred during the compounding process.

In contrast to what is observed for the vast majority of the opaque PC/PMMA blends, in case of the transparent blends the molecular weight distributions of the acetone insoluble parts, i.e., of the fractions consisting of PC and any potentially formed PC-g-PMMA copolymer, in general show strong shifts towards lower molecular weights compared to the pure PC raw material used in the preparation of the blends (Figure 8 and Table 2). This PC molecular weight decrease is particularly severe with SnCl_2_∙2H_2_O. It can be explained by chain scissoring during transesterification according to the mechanism of Singh et al. [6], who proposed a cross-transesterification of a carbonate group in the PC polymer chain with the methyl ester groups in the PMMA side groups. As a consequence of such reaction, the PC molecules involved will be divided into two parts of lower molecular weights that add up to the molecular weight of the initial PC molecule. Only one part will be chemically bonded to a PMMA molecule during the transesterification, while the other will remain as a part of the free PC phase and thus reduce its average molecular weight. However, molecular weight decrease could also be, at least partially, due to hydrolytic degradation of the PC via reaction with residual moisture that was not completely removed by the predrying of the polymer raw materials and/or is introduced by the catalyst (SnCl_2_∙2H_2_O). Our observation that PC molecular weight degradation measured by GPC is also observed upon compounding of the pure PC with the respective catalysts even in the absence of any PMMA, but not to the same extent as observed in the PC/PMMA 50/50 blends (also see Figure 1b), proves that actually both types of reactions likely contribute to the observed total molecular weight decreases. This can also explain why the PC molecular weight decrease is less severe when anhydrous SnCl_2_ is used as catalyst instead of SnCl_2_∙2H_2_O. The latter introduces some additional water into the blend mixture and thus can increase the contribution of hydrolysis reaction to the total decrease of PC molecular weight. The shoulders/second peak at high molecular weight that are observed in the GPC of the acetone insoluble fraction of the transparent PC/PMMA blends are most likely related to the PC-g-PMMA copolymer formed during reactive extrusion (containing both contributions of the PMMA with M_w_=130.000 g/mol and of the PC with M_w_ = 46.000 g/mol). While these GPC contributions of the PC-g-PMMA copolymer in samples (3) and (8) are visible as shoulders, in sample (2) it appears as a well resolved second peak because of the more severe degradation of the PC in this case.

The M_peak_ values of the acetone insoluble fractions of the opaque PC/PMMA blends (Table 2) show no or significantly smaller shifts towards lower levels compared to the transparent blends. The only exception in this context is the blend produced with zinc acetate, which results in a similar molecular weight reduction as the phosphonium salt with which a transparent blend was achieved. The exceptionally severe PC degradation observed with zinc acetate can explain the also exceptional observation of only a single *T_g_* in the DSC of this blend (see above). Obviously, the zinc acetate does catalyze the hydrolytic cleavage of the PC by residual water, but not the transesterification of the PC with PMMA. Thus, the blend produced with zinc acetate is opaque due to absence of PC-g-PMMA copolymer, but the *T_g_* of the PC is reduced to a value similar to that of the PMMA, so that both glass transitions cannot anymore be resolved in the DSC experiment.

#### 3.2.4. Mechanical Performance of the Reactively Compatibilized PC/PMMA Blends

Because effective reactive compatibilization of PC/PMMA blends resulted not only in the targeted formation of PC-g-PMMA copolymer, but inherently also in PC M_w_ degradation as an undesired side-effect, we assessed the mechanical properties of the produced blends to investigate the impact of the combination of both effects on the overall technical performance of the materials. While the formation of PC-g-PMMA copolymer is hoped to result in improved mechanical properties due to phase morphology stabilization, the PC degradation is expected to drive performance in the opposite direction. Previous investigations so far had not reported any mechanical properties of the produced transparent PC/PMMA blends, because the discontinuous lab-scale compounding did not provide sufficient material to do so. In order to allow testing of material properties in accordance with industrially relevant DIN EN ISO standards, we had to scale up the reactive extrusion process to a technical scale to allow production of material quantities sufficient for injection molding of standardized test specimens. For this purpose, a 26 mm twin-screw extruder (ZSK26 MC-18) with a throughput of 20 kg/h and a residence time of 30 s was used to produce PC/PMMA blends in absence of a catalyst as well as in presence of 0.05 wt% SnCl_2_∙2H_2_O, zinc acetate (previously reported benchmarks) and the phosphonium salt. All property data determined on the constituent PC and PMMA raw materials as well as the three produced PC/PMMA 50/50 blends are summarized in Table 3. The different visual phenomenological behaviors of the three blends (level of transparency and color) previously observed on these blends when produced on a laboratory-scale extruder were 1:1 reproduced in this scale-up.

The mechanical, thermal, and rheological properties of the PC/PMMA 50/50 blend produced in absence of any catalyst as expected are all in between the according properties of the constituent polymers. Most of these properties, however, are more or less shifted from the numeric average of both polymer blend constituents to the level observed for PMMA, i.e., the blend behaves more similar to PMMA than might be expected at the 50/50 polymer ratio. The only exception is the tensile strain at yield, which, within accuracy of the measurement, for the PC/PMMA blend is at the same level as for the PC raw material. The blend produced with zinc acetate as catalyst, not only with respect to the visual (optical) properties, but also with regards to the other technical properties, behaves quite similar to the blend produced in absence of any catalyst. The minor differences in terms of melt viscosity and Vicat B/120 are related to the PC M_w_ degradation observed with use of this catalyst (see above). The high haze values of the blends produced in absence of a catalyst (99.4%) and in presence of zinc acetate (98.7%) confirm the visual impression of complete opacity of these materials. The haze determined for the two blends produced with transesterification-effective catalysts also confirm the visual impression of transparency. But moreover, it shows that the phosphonium catalyst results in a more optically clear material compared to the reference catalyst SnCl_2_∙2H_2_O, which, based on this optical measurement, rather has to be considered a translucent than a transparent material. The optical measurements (yellowness index) also confirm the superior color of the transparent blend produced with the phosphonium salt.

Both transparent blends unfortunately show a dramatic deterioration of the mechanical performance in terms of toughness related properties (impact strength, tensile strain at break, tensile strain at yield, and tensile strength) versus the opaque blend of same polymer composition produced in absence of any catalyst. Impact strengths of these two blends fall down to a level that is similar to the pure PMMA as the more brittle blend partner. Tensile strengths, strains at break, and strains at yield fall even below the values of both constituent blend partners. The same is true for the melt viscosity. The heat resistance (Vicat B/120) is negatively affected by the addition of the two catalysts as well. The effects on melt viscosity, heat resistance, and mechanical performance can all be regarded as a direct consequence of the PC M_w_ degradation. Obviously, the detrimental effect of PC M_w_ degradation overcompensates any potentially positive effect of the PC-g-PMMA copolymer formation, which is thus masked. PC/PMMA blends of that low ductility, as observed for the transparent materials obtained via reactive compatibilization, for sure have to be regarded as technically unsuitable for any industrial applications. Thus, different manufacturing process strategies that do not inherently result in PC degradation as a side effect of PC-g-PMMA copolymer formation have to be developed to potentially achieve transparent PC/PMMA blends with a useful balance of properties.

#### 3.2.5. Structure–Properties Relationships—The Root Cause of Transparency of PC/PMMA Blends

**Figure 9:**
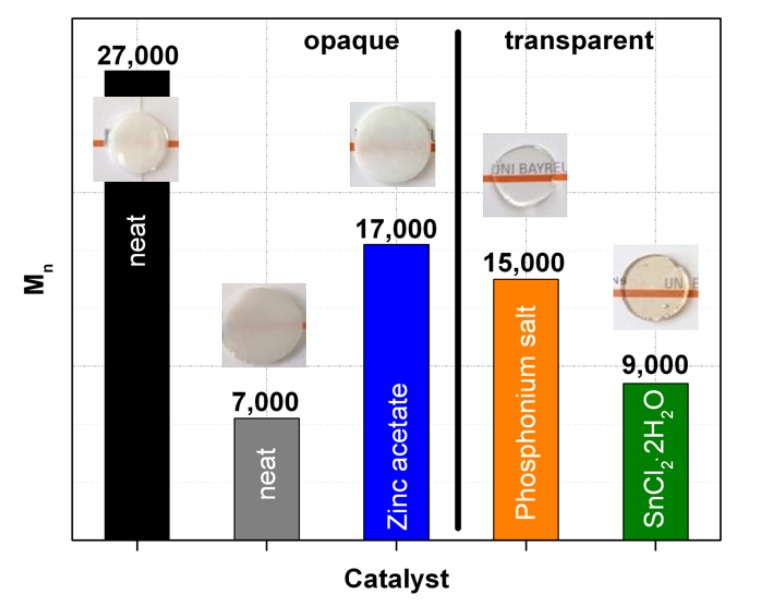
Illustration of the correlation of the haze and the molecular weights (numbers above the columns represent the according values of M_n_) of the acetone insoluble fraction of PC/PMMA 50/50 blends produced in absence of a catalysts (neat) or in presence of 0.05 wt% of zinc acetate, phosphonium salt, and SnCl_2_⋅2H_2_O. FTIR investigation of the acetone insoluble fractions of the blends produced with phosphonium salt and SnCl_2_⋅2H_2_O had proven the presence of PC-g-PMMA copolymer in these materials, while all other blends shown here did not contain any such potential compatibilizer. The grey bar represents a PC/PMMA 50/50 blend produced in absence of a catalyst with the same PMMA as used in the other blends, but with a PC raw material of lower molecular weight.

Figure 9 recaps in form of a graphical illustration the correlation between transparency (haze) and the number averaged PC molecular weight (M_n_) of the acetone insoluble portions of selected PC/PMMA 50/50 blends produced in absence of any catalyst (opaque), and in presence of 0.05 wt% of catalysts zinc acetate (opaque), phosphonium salt (transparent), and SnCl_2_⋅2H_2_O (transparent).

The PC molecular weights of all transparent PC/PMMA 50/50 blends obviously fall below a certain threshold limit of around 15,000 g/mol and, at the same time, contain PC-g-PMMA copolymer that could act as a phase compatibilizer. The opaque blend produced with zinc acetate as catalyst exhibits about the same PC molecular weight as the blend produced with phosphonium salt, but due to the ineffectiveness of the zinc acetate for transesterification does not contain any PC-g-PMMA copolymer. This finding shows that the PC molecular weight reduction alone is not sufficient to result in transparent PC/PMMA blends and thus supports the interpretation that the low PC molecular weight resulting from transesterification is not the root cause (at least not the sole root cause) of the transparency. In order to confirm this conclusion, we designed an additional experiment targeting production of a PC/PMMA blend with a very low molecular weight PC that definitely does not contain any PC-g-PMMA copolymer. For this experiment, a tailor-made polycarbonate feedstock was synthesized that exhibited a molecular weight (M_w_ = 16,700 g/mol, M_n_ = 7000 g/mol) comparable to the lowest level achieved in the reactively compatibilized PC/PMMA compounds (i.e., in presence of SnCl_2_⋅2H_2_O as catalyst) and, like the standard Makrolon^®^ 2408 polycarbonate feedstock used in the other experiments, exhibited a negligible phenolic hydroxyl (pOH) group content, as determined by ^1^H-NMR spectroscopy, of <100 ppm. This tailor-made low molecular weight PC feedstock was compounded with the standard PMMA grade (Plexiglas^®^ 8H)—both were thoroughly predried in vacuum at 60 °C—in the absence of any catalyst to produce a purely physical PC/PMMA 50/50 blend (gray column in Figure 9). The blend turned out opaque, proving our previous conclusion that transparency cannot be exclusively the consequence of the PC M_w_ degradation, i.e., presence of the PC-g-PMMA copolymer which, in our case is the result of a transesterification, is at least a necessary requirement to achieve transparent blends. The question if transparency of PC/PMMA blends can be also achieved in presence of higher molecular weight PC in the resulting blend, or rather transparency requires a combination of both presence of PC-g-PMMA copolymer and a low M_w_ PC cannot be clearly answered based on the results of our current study. This is because with the currently investigated catalytic reactive compatibilization approach, we have not yet succeeded to produce any PC/PMMA blend in which PC-g-PMMA copolymer had been formed to any significant extent and in which the PC molecular weight was significantly higher than the M_n_ = 15,000 g/mol threshold. Starting from Makrolon^®^ 3108 PC feedstock with higher molecular weight than Makrolon^®^ 2408 failed to achieve this target. However, a reliable answer on the above question is crucial to allow a conclusion if transparent PC/PMMA blends with useful mechanical performance are at all technically feasible. Different manufacturing process strategies that do not inherently result in PC degradation as a side effect of PC-g-PMMA-copolymer formation therefore have to be developed and assessed to eventually conclude on this topic.

### 3.3. Mechanism of Transesterification

**Figure 10:**
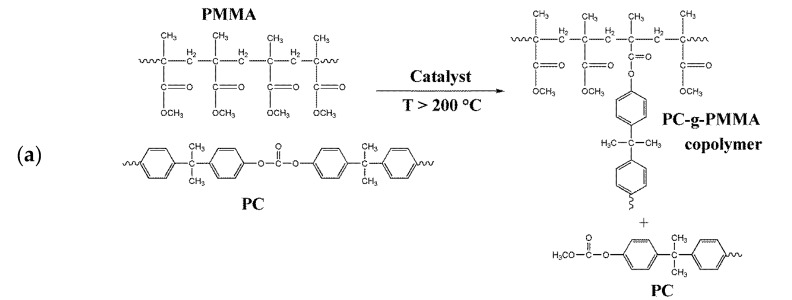

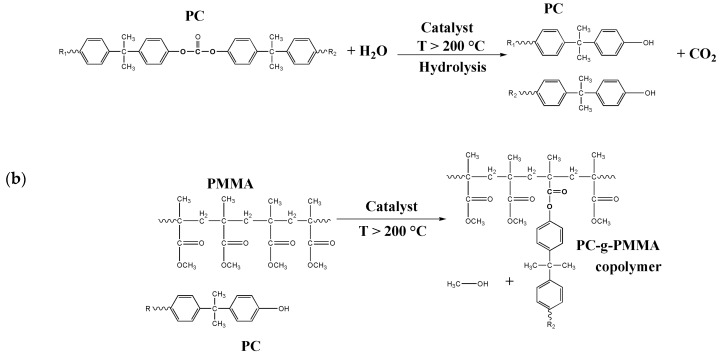
(**a**) One-step cross-transesterification mechanism resulting in PC-g-PMMA copolymer formation as proposed by Singh et al. [6]. (**b**) Two-step hydrolytic scissoring-transesterification mechanism resulting in PC-g-PMMA copolymer formation proposed based on the results of the current study.

Two different mechanisms for transesterification during reactive extrusion for PC/PMMA blends are mentioned in literature. The first one was proposed by Montaudo et al. [18] and assumes an initial depolymerization of the PMMA [18]. The second one has been proposed by Singh et al. [6] more recently and postulates a one-step cross-transesterification reaction of a carbonate group in the PC polymer chain with the methyl ester groups in the PMMA side groups (Figure 10a). Based on our findings of a severe PC M_w_ degradation observed in the presence of the active catalysts even in the absence of any PMMA, we were driven to speculate about a rather two-step transesterification mechanism. In the first step, a hydrolytic chain scissoring of the polycarbonate by residual moisture results in lower M_w_ polycarbonate molecules with phenolic OH end groups. In the second step, a transesterification reaction occurs in which the phenolic hydroxyl end groups of the hydrolyzed PC undergo a nucleophilic attack of the methyl ester side groups of the PMMA (Figure 10b).

**Figure 11:**
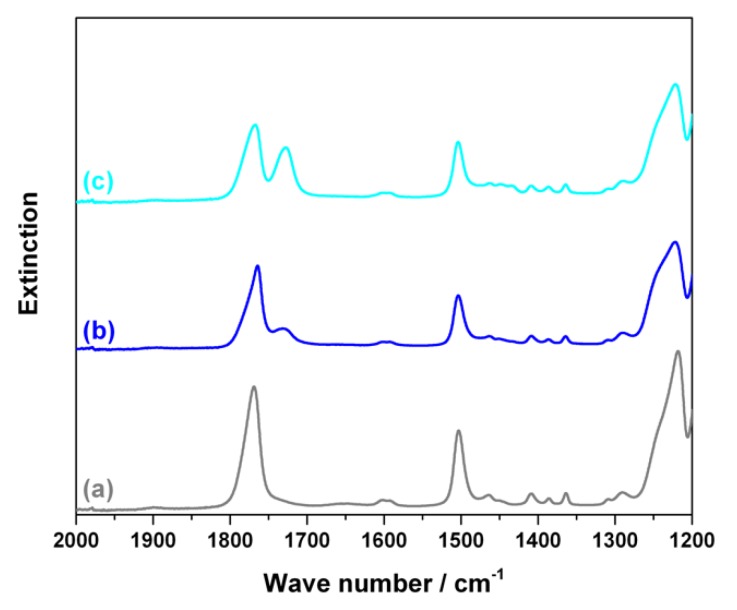
FTIR spectra of the acetone insoluble parts of the PC/PMMA 50/50 blends produced with non-pre-dried polymers in absence of a catalyst (a) as comparison and of the acetone insoluble portions of the PC/PMMA 50/50 blends produced in presence of 0.05 wt% of phosphonium salt (8) based on vacuum-pre-dried (b) and non-pre-dried polymer raw materials (c) (all blends produced on a technical scale extruder ZSK26 MC18).

Targeting verification of this hypothesized transesterification mechanism, we performed additional compounding experiments on the ZSK26 MC18. PC/PMMA 50/50 blends were produced in presence of 0.05 wt% of phosphonium salt starting from PC and PMMA raw materials that, in the first experiment, were used without prior treatment (i.e., equilibrated in ambient air) and in a second experiment were predried in vacuum at 60 °C for 4 h. The FTIR spectra of the acetone insoluble portions of the produced PC/PMMA blends prove that a higher content of PC-g-PMMA copolymer has formed in the experiment in which the raw materials have not been dried prior to compounding, i.e., have contained a higher content of residual moisture (Figure 11). This finding cannot be explained by the one-step cross-transesterification mechanism as proposed by Singh et al. [6] and thus is strong support of the two-step mechanism depicted in Figure 10b postulated here.

## 4. Conclusions

In this study, using a weakly acidic phosphonium salt catalyst, a fully transparent, colorless PC/PMMA 50/50 blend could be produced via catalytic reactive compatibilization of the constituent polymers during compounding in a technical scale twin-screw extrusion process, which can be easily transferred into industrial scale. By doing so, for the first time sufficient material was produced to allow, in accordance with industrially relevant DIN EN ISO standards, assessment of technical properties that are regarded of importance for targeted industrial applications of the blend materials. Extensive characterization of the produced materials at molecular level provided evidence of formation of PC-g-PMMA copolymer as a result of a transesterification reaction occurring during compounding in the presence of those catalysts that resulted in transparent PC/PMMA blends. However, it also demonstrated significant degradation of the polycarbonate molecular weight as an undesired inherent side effect of this transesterification reaction.

Based on compounding experiments using raw materials that contained different levels of residual humidity, the molecular mechanism of this transesterification was refined. In contrast to the previously postulated one-step cross-transesterification of a carbonate group in the PC polymer chain with the methyl ester groups in the PMMA side groups, our results rather propose a two-step mechanism. In a first step the polycarbonate is reacting with residual water under formation of lower M_w_ polycarbonate molecules with phenolic hydroxyl end group (hydrolytic chain scissoring). In a second step, these hydroxyl end groups enter into a transesterification with the methyl ester side groups of the PMMA. Our investigations show that presence of PC-g-PMMA copolymer is required to obtain transparent PC/PMMA blends. Still unclear is if the degradation of the polycarbonate is a secondary necessary requirement for achieving such transparency, since the currently investigated transesterification strategy always resulted in a combination of both M_w_ decrease and copolymer formation. Due to detrimental influences of the PC M_w_ degradation over-compensating any potentially positive impact of the PC-g-PMMA copolymer formation, the reactive compatibilization results in a dramatic deterioration of the toughness related mechanical properties of the produced transparent blends, which makes them technically unsuitable for any industrial applications. Within ongoing activities, we are investigating different approaches of PC-g-PMMA copolymer formation that do not result in PC degradation in order to allow a conclusion if transparent PC/PMMA blends with a useful balance of properties are technically feasible.

## 5. Patents

A patent application has been filed on April 18, 2019 regarding use of the proprietary, weakly acidic phosphonium salt that has been investigated in this study in reactive extrusion processes in a twin-screw extruder involving polycarbonate. Because the patent application has not yet been published at the time of submission of this paper we cannot disclose the exact nature of the phosphonium salt, since this would potentially interfere with the interests of the patent applicant. For future reference, please see:

A. Seidel, T. Bubmann (inventors), Covestro Deutschland AG (applicant), EP patent registration (filing number: 19170040.0) “Katalysator für Reaktivextrusion in einem Doppelwellenextruder unter Einsatz von Polycarbonat”.

## Figures and Tables

**Table 1:** Used transesterification catalysts.

Catalyst	Sample Denotation (Number)	Form	Supplier
No catalyst Tin(II) chloride dihydrate (SnCl_2_∙2H_2_O)	1 2	Powder	Merck (Darmstadt, Germany)
Tin(II) chloride anhydrous	3	Powder	Fisher Scienfic (Hampton, NH, USA)
Tin(II) acetate	4	Powder	Alfa Aesar (Haverhill, MA, USA)
Tin(II) ethyl hexanoate	5	Liquid	Alfa Aesar (Haverhill, MA, USA)
Tin(II) acetylacetonate	6	Liquid	VWR International (Radnor, PA, USA)
Zinc acetate	7	Powder	Merck (Darmstadt, Germany)
Phosphonium salt	8	Powder	-
Zinc(II) acetylacetonate	9	Powder	Sachem, Inc. (Austin, TX, USA)

**Table 2:** Molecular weights at the peak position of the M_w_ distribution (M_peak_) of the acetone insoluble parts of the transparent and opaque PC/PMMA 50/50 compounds.

Sample	Catalyst	M_peak_ [kg/mol]
**Reference**	-	
PC raw material		46
**Transparent compounds**		
Sample 2	SnCl_2_∙2H_2_O	10
Sample 3	SnCl_2_	26
Sample 8	phosphonium salt	26
**Opaque Compounds**	-	
Sample 1		46
Sample 4	Tin(II) acetate	40
Sample 5	Tin(II) ethyl hexanoate	45
Sample 6	Tin(II) acetylacetonate	41
*Sample 7*	*Zinc acetate*	*28*
Sample 9	Zinc(II) acetylacetonate	35

**Table 3:** Technical properties of the constituent PC and PMMA polymers as well as of the PC/PMMA (50/50) blends produced therewith in absence of a catalyst and in presence of 0.05 wt% of phosphonium salt, SnCl_2_⋅2H_2_O and zinc acetate (all blends produced on a technical scale extruder ZSK26 MC18).

Material Property	PC neat	PMMA neat	PC/PMMA 50/50	PC/PMMA 50/50	PC/PMMA 50/50	PC/PMMA 50/50
	-	-	-	Phosphonium Salt 0.05 wt%	SnCl_2_⋅2H_2_O 0.05 wt%	Zinc Acetate 0.05 wt%
**Haze [%]**	0.3	0.4	99.4	0.5	11.4	98.7
**Yellowness index**	2.4	0.4	40	1.8	8.2	48
**Tensile modulus [MPa]**	2175	3167	2748	2838	2875	2799
**Tensile strength [N/mm^2^]** **Tensile strain at yield [%]** **Tensile strain at break [%]**	63 6.5 109	91 5.9 6	82 6.6 57	52 n.y. ** 2	52 n.y. ** 2	80 6.5 55
**Impact strength [kJ/m^2^]**	n.b.*	19	37	18	21	29
**Vicat B/120 [°C]**	149	111	125	120	121	123
**Melt viscosity (260°C/1000s^−1^) [Pas]**	606	232	296	137	147	230

* n.b. = no break; ** n.y. = no yield.
